# Is it all about the self? The effect of self-control depletion on ultimatum game proposers

**DOI:** 10.3389/fnhum.2013.00240

**Published:** 2013-06-13

**Authors:** Eliran Halali, Yoella Bereby-Meyer, Axel Ockenfels

**Affiliations:** ^1^Department of Psychology, Ben-Gurion University of the NegevBeer-Sheva, Israel; ^2^Department of Economics, University of CologneCologne, Germany

**Keywords:** social preferences, fairness, ultimatum game, dictator game, dual process, cognitive-control, self-control, ego-depletion

## Abstract

In the ultimatum-game, as in many real-life social exchange situations, the selfish motive to maximize own gains conflicts with fairness preferences. In the present study we manipulated the availability of cognitive-control resources for ultimatum-game proposers to test whether preference for fairness is a deliberative cognitive-controlled act or an automatic act. In two experiments we found that a shortage of cognitive control (ego depletion) led proposers in the ultimatum game (UG) to propose significantly more equal split offers than non-depleted proposers. These results can be interpreted as resulting from an automatic concern for fairness, or from a greater fear of rejection, which would be in line with a purely self-interested response. To separate these competing explanations, in Experiment 2 we conducted a dictator-game in which the responder cannot reject the offer. In contrast to the increased fairness behavior demonstrated by depleted ultimatum-game proposers, we found that depleted dictator-game allocators chose the equal split significantly less often than non-depleted allocators. These results indicate that fairness preferences are automatically driven among UG proposers. The automatic fair behavior, however, at least partially reflects concern about self-interest gain. We discuss different explanations for these results.

## Introduction

Behavioral decision-making research suggests that behavior is best understood as resulting from the operation of at least two underlying systems: the affective (system 1) and the deliberative (system 2). The affective system is generally described as fast, automatic, associative in nature, emotionally charged, and requires minimal cognitive resources. In contrast, the deliberative system is slow, deliberately controlled, analytical, affect free, and requires cognitive resources (e.g., Stanovich, 1999; Kahneman and Frederick, 2002, pp. 49–81; and for an overview: Evans, 2008). For individual decision-making tasks, such as inter-temporal choice, agreement exists among researchers about the behavior expected under the affective system, but for social decision-making the evidence is equivocal (e.g., Loewenstein et al., 2008). It is not clear whether economic self-interest or social preferences, such as fairness, are the primary motives (i.e., automatic) that need to be controlled by the deliberative system. In the current study, we contribute to this ongoing discussion by studying the role of cognitive-control in fairness behavior. Specifically, we examine whether fairness behavior is a deliberate act that requires self-control or whether it is evoked automatically. Answering this question is important because people often make social decisions under conditions of limited cognitive-control resources, such as exhaustion, sleep deprivation, cognitive load, and time pressure.

A well-known paradigm customarily used to study fairness perception and behavior is the Ultimatum Game (UG; Guth et al., 1982). In this game, two players are given an opportunity to split a sum of money. One player proposes how to split the sum, and another player responds. If the responder accepts the offer, the money is split as proposed. If the responder rejects the offer, neither player receives anything. The standard economic model dictates that the proposer should offer the smallest possible amount of money since the responder would accept any offer above zero. Contrary to this prediction, empirical results show that individuals consider fairness in their offers and choices. Proposers, on average, ask for less than 70% of the total sum, and responders usually reject unfair offers (for an overview: Camerer, 2003).

Models of social preferences address this fairness behavior. According to inequality aversion theories, people may not only care about their absolute outcome but also about their relative share (e.g., Fehr and Schmidt, 1999; Bolton and Ockenfels, 2000). As a result, people may prefer to decrease the difference between their outcome and the outcome for others, even if this diminishes their absolute outcome. Alternatively, according to reciprocal fairness based theory, people care about the intention behind the offer and are willing to pay to punish (or reward) their opponents for their unfair (fair) offers (e.g., Rabin, 1993; Blount, 1995; Dufwenberg and Kirchsteiger, 2004; Bereby-Meyer and Niederle, 2005; Radke et al., 2012).

It has been suggested that fairness preferences result from deliberation processes (Moore and Loewenstein, 2004; see Knoch et al., 2006, for neurological support among UG responders). According to this view, egoism-based self-interest is the primary motive that needs to be constrained. In line with this suggestion, developmental studies have found that kindergarteners behave according to the standard economic model (e.g., Bereby-Meyer and Fiks, in press), while fairness preferences are most likely learned throughout life (Güroğlu et al., 2009, 2011; Bereby-Meyer and Fiks, in press). However, the majority of neurological (e.g., Sanfey et al., 2003; Tabibnia et al., 2008) and behavioral (e.g., Cappelletti et al., 2011; Halali et al., in press) findings regarding UG responders suggest that by adulthood reciprocal fairness preferences become automatic relative to self-interest considerations. Thus, they are those that need to be controlled. Accordingly, Halali et al. (in press), found that a shortage of cognitive-control resources resulted in an increase in rejection rates of unfair offers in the UG, i.e., an increase in reciprocity behavior.

### Current research

In the current study, we examine the effect of cognitive control shortage on fairness behavior of UG proposers. By cognitive control (also termed “self-control” or “executive-control”; e.g., Schmeichel, 2007; Robinson et al., 2010) we mean the ability to “deliberately inhibit dominant, automatic, or prepotent responses,” in order to maximize the long-term best interests of the individual (e.g., Mischel, 1996; pp. 197–218; Muraven and Baumeister, 2000). According to the deliberative approach to fairness preferences (e.g., Moore and Loewenstein, 2004) self-interested behavior will increase under a shortage of cognitive control, i.e., an increased rate of unfair UG offers is expected. Contrary to that prediction, based on the automatic tendency of reciprocal fairness observed in the UG responders' behavior (Halali et al., in press), we expect an increase in fairness behavior under a shortage of cognitive control. Initial support for this hypothesis can be found in Rubinstein (2007) who found that equal split offers compared to non-equal offers are implemented faster, and by Cappelletti et al. (2011) who found that UG proposers offer more under time pressure.

To reveal the automatic tendency of UG proposers, in the current study, following Halali et al. (in press), we adopted the strength model suggested by Baumeister et al. (1998). According to this theory, self-control relies on a limited resource that gets depleted when one tries to inhibit competing behaviors, urges, or desires, just as a muscle tires after performing an effortful action. Consequently, an initial act of self-control impairs subsequent acts of self-control, even in unrelated tasks; this state is called *ego-depletion* (Baumeister et al., 1998; Muraven et al., 1998; Vohs and Heatherton, 2000; for a review, see Baumeister et al., 2007). The limited resource explanation has been disputed recently (e.g., Inzlicht and Schmeichel, 2012), however, there is agreement regarding the ego-depletion phenomenon, given the numerous experiments that support this finding (for a meta-analysis: Hagger et al., 2010). Thus, given that deliberate actions require cognitive-control resources, a state of depletion should increase automatic behavior (e.g., Vohs, 2006; Masicampo and Baumeister, 2008). In two experiments, we had our participants undergo an ego depletion manipulation and then examined the (un)fairness of their offers in the role of proposers in the UG. Given our assumption that fairness preferences are automatic, we expected an increased rate of fair offers by depleted participants compared to non-depleted participants.

## Experiment 1

### Method

#### Participants

Twenty nine participants (14 Female and 15 Male) with no previous knowledge of the UG, participated in exchange to 20 New Israeli Shekels (NIS; approximately $5). We informed participants ahead of time that we will randomly choose five participants and pay them according to their actual earnings in one random trial of the UG, which we actually did. We randomly assigned participants to one of two experimental conditions: depletion (*n* = 14; 7 Females, 7 Males), no-depletion (*n* = 15; 7 Females, 8 Males).

### Materials

#### Depletion task

We manipulated the cognitive-control resources depletion using Mead et al.'s 2009) procedure, which has been also used by Halali et al. (in press). Participants in the *depletion condition* completed 20 incongruent trials of the Stroop (1935) task. In each trial, participants had to name the color of the ink and suppress their automatic tendency to read the incongruent color word. In the *no-depletion condition*, the words matched the ink colors, making it unnecessary to ignore the words. Therefore, the incongruent condition required more cognitive-control resources than did the congruent condition.

#### UG task

**Figure 1:**
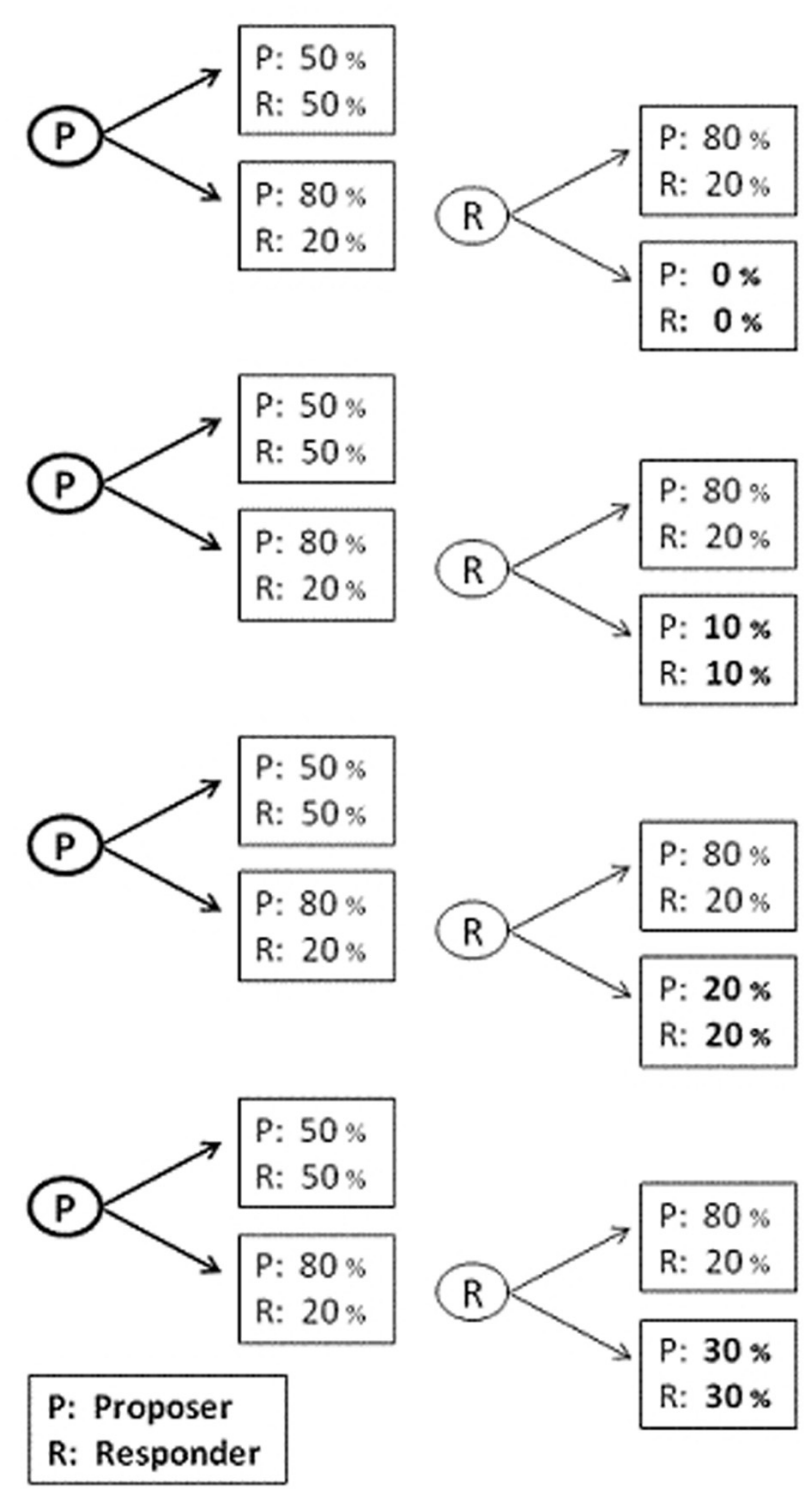
**Experiment 1's UG task with four different Rejection-Outcome**.

We randomly assigned participants to the role of proposers in a computerized version of a mini UG. We first thoroughly instructed participants about the nature of the rules of the UG. The task included 8 different independent trials with 8 different responders, who play the game in a different session of the same experiment. Other than that, we did not give the participants any other information regarding the responders. In each round, proposers had to make a one-time monetary offer of either a fair division, i.e., 50% of the stake for both players, or an unfair division, i.e., 80% of the stake to the proposer and 20% to the responder. Four different “Rejection-Outcomes” were associated with the different offers. As can be seen in Figure 1, these outcomes were: 0, 10, 20, or 30% of the stake to each player. For each Rejection-Outcome we implemented two different “Stake-Size”: 100 NIS and 200 NIS (~25 and $50, respectively). We presented the 8 trials (4 Rejection Outcome ×2 Stake-Size) in a random order. We randomized the location of the equal split (50:50) on the screen (i.e., left/right) and its response-key within participants. To avoid outcome effects we did not give participants feedback about the responders' choices during the experiment. Note that the higher the Rejection-Outcome is, the lower are the consequences of a rejection for proposers' payoff. This tendency, however, is the same for the responders, which causes the risk of rejection to increase. Consequently, we do not expect the Rejection-Outcome to affect proposers' offers or to interact with the experimental condition. Thus, we were able to improve the statistical power of our test by presenting participants with several repetitions of the UG while minimizing the risk that participants will be bored.

#### Mood and arousal

Participants completed the Brief Mood Introspection Scale (BMIS; Mayer and Gaschke, 1988) that measures mood and arousal. The BMIS assesses participants' current mood based on their responses to 16 adjectives. In particular, participants rate how they feel in relation to each of the adjectives on a five-point Likert scale (1 = definitely do not feel, 5 = definitely do feel). The scale contains two subscales; mood valance and arousal.

### Procedure

We invited participants to different timeslots in groups of up to six participants each. With arrival participants were seated in a separate computer desks. All the assignments and questionnaires were computerized. After signing the consent form, and before starting the depletion task, we verbally informed participants that they would participate in a number of separate and independent experiments. Following the Depletion task, participants reported their mood and arousal levels on the BMIS, and following the UG task, they answered a questionnaire aimed at assessing suspicion regarding their partners in the UG. Participants had to indicate regarding the identity of the responders whether they are: “participants (a) from different sessions; (b) in the same lab with them; (c) different: ______.”

### Results and discussion

Two participants were excluded from the analysis since they indicated they did not believe they are playing a real game with real responders. The pattern of the following reported results was the same when these participants were included in the analysis.

We submitted the participants' offers (0: unequal, 1: equal split offers) to a three-way generalized probit estimation equations for binominal data with Condition (depletion, no-depletion) as a between-participant independent variable, Rejection-Outcome (0, 10, 20, 30%) and Stake-Size (100, 200 NIS) as within-participant independent variables, and the participants as a random factor.

We found a significant main effect for Condition: *Wald* χ^2^(1) = 4.12, *p* = 0.042. As expected, on average, compared with the no-depletion condition (*M* = 52.7%), depletion state resulted in significantly higher rate of equal split offers (*M* = 68.3%). In addition, a two way interaction between Rejection-Outcome and Stake-Size was found, (*Wald* χ^2^(3) = 9.39, *p* = 0.025). Since we did not have a clear prediction regarding this interaction and it was not relevant to the significant main effect of Condition which is the focus of this experiment, we did not further analyze this interaction. No other effect or interactions were significant (all *p*s > 0.05).

The experimental condition did not affect Mood (no-depletion: *M* = 15.0, *SD* = 6.9, depletion: *M* = 12.1, *SD* = 6.9; *F* < 1.2, *n.s*.) or Arousal (no-depletion: *M* = 16.4, *SD* = 4.7, depletion: *M* = 17.4, *SD* = 6.0; *F* < 1, *n.s*.), thus, they are unlikely to account for the reported effect.

To summarize, the results of Experiment 1 showed that a shortage of cognitive-control resources led to an increase in the tendency to propose fair offers in the UG. This increase in fair offers proposals is in line with the tendency of UG proposers to propose more fair offers under time pressure (Cappelletti et al., 2011). In principle, the observed high proportion of fair offers may either be due to an automatic fear of rejection (selfish component) or to automatic fairness preferences. In Experiment 2, we tried to decide between these two motives.

## Experiment 2

To further disentangle the two aforementioned explanations for the automatic tendency to propose fair offers in the UG, in Experiment 2 we use the dictator game (DG; Forsythe et al., 1994), a variant of the UG in which the responder cannot reject the offer. The advantage of the DG is that there is no fear of rejection in this game. At the same time, however, the DG removes the reciprocal relationship inherent in the UG—an observation that we get back to in the concluding section. If fairness preferences are automatic then we expect in the UG as well as in the DG to an increase in the rate of fair offers under a shortage in cognitive control. However, if selfish considerations associated with the perceived risk drive the automatic tendency to propose higher offers, then, we don't expect an increase in fair offers in the DG under a shortage in cognitive control. If something, we may even expect a decrease in the rate of fair offers in the DG. This prediction will be in line with the reduction in helping behavior observed among depleted participants (DeWall et al., 2008; Xu et al., 2012), and with a recent response-time study on DG allocators (Piovesan and Wengström, 2009) which found that self interested choices are made quicker than fair choices, both in a between and a within participants analysis.

Finally, one of the major dispositional factors related to decision-making in social situations is social value orientation (SVO; Van Lange et al., 1997). SVO are individual differences in how people evaluate outcomes for themselves and others (Messick and McClintock, 1968; Kuhlman and Marshello, 1975). Van Lange (1999) suggested that most people can be classified as being pro-socials, competitors, or individualists. Because individualists and competitors—both assign a higher weight to their own outcomes than to the outcomes of others they are usually taken together and defined as pro-self (e.g., Van Lange and Kuhlman, 1994). Regarding fairness preferences, Van Dijk et al. (2004) found that only pro-self participants are sensitive to the strategic aspect of the UG game. For example, in one of their experiments, when responders received incomplete information, pro-self participants took advantage of that and kept for themselves more money, while leading the responders to believe that they got a fair proposal. Yet, Van Dijk et al. (2004), have not examined the effect of SVO on automatic fairness behavior. In Experiment 2, therefore, we also assessed participants' SVO using the decomposed games measure suggested by Van Lange et al. (1997).

### Method

#### Participants

One hundred and seventeen undergraduate students (59 Female and 58 Male) with no previous knowledge on the UG or the DG, participated in exchange to extra course credit. We randomly assigned participants to one of four experimental conditions: UG depletion (*n* = 24; 11 Females, 13 Males), UG no-depletion (*n* = 25; 14 Females, 11 Males), DG depletion (*n* = 37; 18 Females, 19 Males), DG no-depletion (*n* = 31; 16 Females, 15 Males). We informed participants ahead of time that we will randomly choose 8 participants and pay them according to their actual earnings in one random trial of the UG/DG, which we actually did.

### Materials

#### Depletion task

We manipulated cognitive-control resources depletion using the Schmeichel's 2007) procedure. We instructed participants in the *no-depletion condition* to “Write a story about a recent trip you have taken. It may be a trip to a store, to some location in Israel, or to another country—wherever! Please keep writing until the computer program asks you to stop.” For participants in the *depletion condition* we gave an additional instruction: “Very important! Please do not use the letters “*Aleph*” (equivalent to the English letter *a*) or “*Nun*” (equivalent to the English letter *n*) anywhere in your story.” Hence, one group was required to regulate their writing by avoiding the use of two common letters, whereas the other group did not get any writing restrictions. The experimenter stopped all participants after 5 min of writing.

#### UG/DG task

First we thoroughly instructed participants about the rules of the game they were assigned to. In the UG participants played in the role of proposers, offering one-time monetary offers to 4 different responders. Each offer involved different stake size: 100, 80, 50, and 20 NIS (~25, 20, 12.5 and $5, respectively), presented in a randomized order. We used a computerized version of the UG, in which the responders are participants from another academic institution who play the game in a different session of the same experiment. Other than that, we did not give the participants any other information regarding the responders. On each trial, participants first saw the stake amount for that trial, and then a response scale indicating the proportion of the stake size that they want to offer to their partner, from 0 to 50%, in increments of 10. In the DG, the task was the same as in the UG except for the fact that the responder has no decision to make.

#### Assessment of social value orientation

As the last task, following an unrelated filler task, participants completed a nine-item Decomposed Games Measure (Van Lange et al., 1997). They chose among combinations of outcomes for themselves and for an anonymous other. These choices are made in a non-strategic setting (i.e., the outcomes depend only on what the participant chooses). Outcomes are represented by points, and participants are instructed to imagine that the points have value to themselves and to the other person. Each option represents a particular orientation. An example is the choice between alternative A: 500 points for self and 100 points for other, B: 500 points for self and 500 for other, and C: 550 points for self and 300 for other. Option A represents the competitive orientation because this distribution maximizes the difference between one's own outcomes and the other's outcomes (Choice A: 500–100 = 400, vs. B: 500–500 = 0, and C: 550–300 = 250). Option B represents the cooperative or pro-social orientation, because it provides an equal distribution of outcomes (i.e., 500 for self and other), and generates the highest number of collective outcomes (i.e., 1000). Finally, option C represents the individualistic option because one's own outcomes are maximized (550 vs. choice A: 500, and B: 500) irrespective of the other's outcomes. Participants are classified as pro-social, individualistic or competitive when at least six choices (out of nine) are consistent with one of the three orientations (e.g., Van Lange and Kuhlman, 1994). As in some prior research on SVO, we combined the individualists and competitors to form a group of pro-self individuals (e.g., Van Dijk et al., 2004).

#### Mood and arousal

We measured mood and arousal using the BMIS, in the same way as in Experiment 1.

### Procedure

We invited participants to different timeslots in groups of up to 6 participants each. Each participant sat in a separate computer desks. All the assignments and questionnaires were computerized. Following the UG/DG task, participants answered a questionnaire that assesses suspicion regarding their partners in the UG/DG. They indicated regarding the identity of the proposers whether they are: “participants (a) from other academic institutes; (b) from future sessions in the same institute; (c) in the same lab with them; (d) different: ______.” Next, as a manipulation check, participants rated the difficulty of the writing task, on a scale from 1 (not at all difficult) to 7 (very difficult), and reported their mood and arousal levels on the BMIS.

### Results and discussion

A total of 23 participants were excluded from all analyses. Fifteen participants were excluded because of their performance in the *depletion regulated-writing task*: 13 participants (5 in the UG, 8 in the DG) used the forbidden letters in over 10% of the words they wrote, and 2 participants (1 in the UG, 1 in the DG) did not write anything at all. Eight participants (3 in the UG and 5 in the DG) were excluded since they indicated they did not believe they were playing a real game with real responders. The pattern of the following reported results was the same when these participants were included in the analyses.

#### Manipulation check

Ratings of the difficulty of the initial writing task indicate that the instructions in the depletion condition indeed were more difficult to follow (*M* = 4.14, *SD* = 1.84) than the free writing instructions in the no-depletion condition (*M* = 2.42, *SD* = 1.49), *F*_(1, 92)_ = 25.04, *p* < 0.001, η^2^_*p*_ = 0.21. This pattern is consistent with the assumption that the two writing instructions required different degrees of cognitive-control.

#### Decision-making

No main effects for Gender or for Stake-Size or any interactions with these factors were found in any of the analyses (all *p*s > 0.05) and thus these factors were not further analyzed.

**Figure 2:**
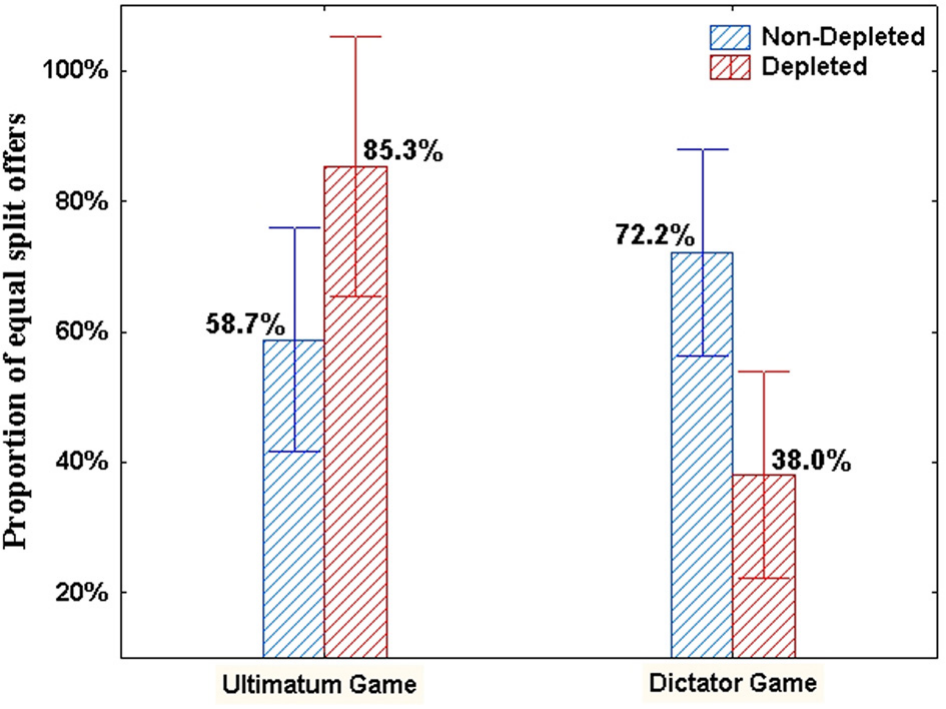
**Means and standard errors of the proportion of equal split offers in Experiment 2 as a function of Game and Condition**.

We calculated for each participant the proportion of equal split offers (i.e., 50% of the stake). We submitted the proportion of equal split offers to a Two-Way ANOVA with Game (UG, DG) and Condition (depletion, no-depletion) as between-participant independent variables^1^. Consistent with previous results regarding the UG and the DG, we found a marginally significant main effect for the Game: *F*_(1, 90)_ = 3.78, *p* = 0.055, η^2^_*p*_ = 0.04, indicating that the proportion of equal split offers was higher for UG participants (*M* = 70.0%, *SD* = 40.9) compared to DG participants (*M* = 55.1%, *SD* = 45.9). While the main effect of Condition was not significant (*F* < 1, *n.s*.), the Game × Condition interaction was highly significant, *F*_(1, 90)_ = 12.25, *p* < 0.001, η^2^_*p*_ = 0.12 (see Figure 2).

To probe the significant interaction, we conducted two simple contrast analyses, one for the UG and one for the DG. In each contrast we compared the average proportions of equal split offers in the depletion and in the no-depletion groups. Consistent with Experiment 1's results, depleted UG participants proposed significantly higher rate of equal split offers (*M* = 85.3%, *SD* = 30.7) compared to the non-depleted participants (*M* = 58.7%, *SD* = 44.3), *F*_(1, 90)_ = 4.04, *p* < 0.05, η^2^_*p*_ = 0.04. The pattern of results was reversed for the DG participants, i.e., depletion state resulted in significantly lower rate of equal split offers (*M* = 37.9%, *SD* = 45.1) compared to the no-depletion state (*M* = 72.2%, *SD* = 40.6), *F*_(1, 90)_ = 9.25, *p* < 0.01, η^2^_*p*_ = 0.09.

The experimental condition, as in Experiment 1, did not affect Arousal as measured by the BMIS (no-depletion: *M* = 20.0, *SD* = 5.6, depletion: *M* = 19.4, *SD* = 5.7; *F* < 1, *n.s*.). Thus, Arousal is unlikely to account for the reported effects. However, for Mood valence (i.e., pleasant vs. unpleasant), we found a main effect of Condition, *F*_(1, 90)_ = 4.75, *p* < 0.05, η^2^_*p*_ = 0.05. Different than expected participants who performed the writing task in the depletion condition reported being in a more pleasant mood (*M* = 10.57, *SD* = 9.61) than participants who performed the free-writing task in the no-depletion condition (*M* = 6.24, *SD* = 9.44), with no main effect for Game nor an interaction between Game and Condition (both *F*s *<* 1*, n.s*.). To rule out the possibility that the mood accounts for the differences in the proposals we repeated the analysis on the proportion of equal split offers, while including as covariates Mood valence and Arousal. Neither Mood (*F* < 1, *n.s*.), nor Arousal (*F* < 1.25, *n.s*.), reliably predict the proportion of equal split offers, whereas the marginally significant main effect for Game [*F*_(1, 88)_ = 3.47, *p* = 0.066, η^2^_*p*_ = 0.04], and the significant Game × Condition interaction [*F*_(1, 88)_ = 10.69, *p* < 0.002, η^2^_*p*_ = 0.11] obtained in the original analysis were hardly affected. Hence, although there were unexpected differences in self-reported Mood valence between the two experimental conditions, these differences did not account for the pattern of results in the UG and the DG tasks.

#### Social value orientation

In the present experiment, out of 94 participants included in the previous analysis, nine participants (4 in the UG, 5 in the DG) made fewer than six consistent choices according to one of the three orientations (e.g., Van Lange and Kuhlman, 1994) in the nine-item Decomposed Games Measure. Hence, they could not be classified and were therefore excluded from further analyses. Of the 85 remaining participants, 51 (60.0%) were classified as pro-social and 34 (40.0%) as pro-self. The distribution of pro-socials and pro-selfs in each experimental condition was as follows: UG depletion (*n* = 15: 10 pro-socials, 5 pro-selfs), UG no-depletion (*n* = 21: 11 pro-socials, 10 pro-selfs), DG depletion (*n* = 27: 16 pro-socials, 11 pro-selfs), DG no-depletion (*n* = 22: 14 pro-socials, 8 pro-selfs). There were no significant differences in the proportion of pro-socials in each of the four experimental conditions (all χ^2^ < 1, *n.s.*).

We repeated the analysis for the proportion of equal split offers with participants' SVO (pro-self, pro-social) as an additional between-participant independent variable. The Game × Condition interaction remained significant, *F*_(1, 77)_ = 10.07, *p* < 0.003, η^2^_*p*_ = 0.12, with the same pattern as previously reported. We also obtained a significant main effect for SVO, *F*_(1, 77)_ = 6.21, *p* < 0.05, η^2^_*p*_ = 0.07, indicating that, across Game and Condition, pro-social participants had a higher rate of equal split offers (*M* = 70.0%, *SD* = 40.3) compared to pro-self participants (*M* = 43.4%, *SD* = 47.8). No other effect was significant (all *p*s *>* 0.10). Therefore, SVO did not moderate the depletion effect for UG or DG proposers.

To summarize, in Experiment 2 we replicated the results of Experiment 1 for UG proposers, using a different manipulation for ego-depletion and a different structure of the game. Specifically, a shortage of cognitive-control resources resulted in an increase of fair behavior. For depleted DG allocators however, we found the reversed pattern, i.e., they demonstrated a decrease of fair behavior compared to non-depleted allocators. Further, in line with previous findings (Van Dijk et al., 2004), pro-social participants tended overall to care more for fairness than pro-self participants. Yet, participants' SVO did not moderate the effect of ego depletion in the UG or in the DG. It is worth noticing, however that the number of participants within each orientation (i.e., pro-self/pro-social) in each condition is relatively small.

## General discussion

Is the automatic fairness tendency of UG proposers due to automatically elicited fairness preferences, or is it due to an increased fear of rejection, i.e., automatic strategic selfish preferences associated with risk perceptions?

The observation in Experiment 2 that depleted DG allocators became more selfish compared to the non-depleted allocators indicates that participants were less concerned with fairness when the fear of rejection was absent. The automatic selfish behavior demonstrated by depleted DG allocators is consistent with the findings of a recent study which demonstrated that ego-depletion reduced the willingness to help others (DeWall et al., 2008). This effect was mediated by decreases in guilt feelings (Xu et al., 2012). Notably, if fairness preferences drive the high proposed offers of depleted UG proposers, we should have observed higher offers from depleted, compared to non-depleted DG allocators as well. Given the reversed observed pattern for depleted DG allocators, the increase in fair behavior of the depleted UG proposers probably reflects an automatic selfish fear of rejection.

Interestingly, the increase in fairness behavior among UG proposers matches the automatic behavior of UG responders documented in most studies on that matter (e.g., Cappelletti et al., 2011; Halali et al., in press). Specifically, it corresponds with the increase in negative reciprocity of UG responders following a shortage of cognitive control resources (Halali et al., in press). The results of the current study, however, suggest that this match in behavior is probably driven by different motivations, namely, depleted UG proposers are motivated by automatic selfish preferences rather than automatic fairness preferences, that probably motivate depleted responders in the UG.

At first glance, given the reasoning aspect assumed to be involved in strategic thinking it sounds contradictory that strategic considerations are revealed under a shortage of cognitive control. We suggest that different types of emotions, which are affected differently by a shortage in cognitive control, may explain this counter intuitive result (Halali et al., in press). Specifically, strategic considerations of UG proposers are driven by fear that their offer will be rejected (e.g., Nelissen et al., 2011). In contrast, fair behavior of DG allocators is suggested to be driven by guilt (e.g., Ellingsen et al., 2010). While fear is an immediate experienced emotion that is viscerally driven (Loewenstein, 1996, 2000) and therefore is dominant under a shortage of cognitive control resources (Wagner and Heatherton, 2013; Vohs et al., submitted), guilt is an anticipated emotion that is likely to be reduced under ego depletion (e.g., Xu et al., 2012). While Nelissen et al. (2011) suggested that guilt is an additional motivation for fair behavior of UG proposers, given the finding that UG offers are in most cases higher than DG offers, the strategic component in the UG (i.e., fear of rejection) is probably more dominant in this game. The current results suggest that this fear of rejection in the UG is even more pronounced under ego depletion.

Another possible explanation for the seemly mixed results for UG proposers and DG allocators may be the characteristics of the games, which may trigger different motives for being fair. Much research has concluded that UG behavior is mainly driven by reciprocity (e.g., Rabin, 1993; Blount, 1995; Dufwenberg and Kirchsteiger, 2004; Bereby-Meyer and Niederle, 2005; Radke et al., 2012)—a motive that cannot explain DG behavior, because the receiver in the DG cannot reciprocate (reward or punish) the allocator's offer. This seems to suggest an alternative hypothesis: in games of reciprocity the automatic response is to behave in a reciprocally fair way, while in games without reciprocal interaction, selfishness is the automatic response. This hypothesis is consistent with automatic fairness on both the proposer's and responder's side in the UG, as well as with automatic selfishness in the DG. Further research is needed to distinguish between these possible explanations.

### Conflict of interest statement

The authors declare that the research was conducted in the absence of any commercial or financial relationships that could be construed as a potential conflict of interest.

## Footnotes

^1^A Probit regression with subjects as a random variable, participants' offers as the dependent variable and Condition, Game and the interaction between them as independent variables, revealed the same results.
